# Characterization of native plant growth promoting rhizobacteria and their anti-oomycete potential against *Phytophthora capsici* affecting chilli pepper (*Capsicum annum* L.)

**DOI:** 10.1038/s41598-020-69410-3

**Published:** 2020-08-17

**Authors:** Sajjad Hyder, Amjad Shahzad Gondal, Zarrin Fatima Rizvi, Raees Ahmad, Muhammad Mohsin Alam, Abdul Hannan, Waqas Ahmed, Nida Fatima, M. Inam-ul-Haq

**Affiliations:** 1 Department of Plant Pathology, PMAS Arid Agriculture University, Rawalpindi, Pakistan; 2 Department of Botany, G.C Women University, Sialkot, Pakistan; 3 Department of Plant Pathology, Bahauddin Zakriya University, Multan, Pakistan; 4 Department of Botany, Ghazi University, D. G. Khan, Pakistan; 5 Department of Soil Science and SWC, PMAS Arid Agriculture University, Rawalpindi, Pakistan

**Keywords:** Biotic, Microbe, Biotic, Microbe

## Abstract

*Phytophthora capsici* is a notorious fungus which infects many crop plants at their early and late growth stages. In the present study, twelve *P. capsici* isolates were morphologically characterized, and based on pathogenicity assays; two highly virulent isolates causing post-emergence damping-off on locally cultivated chilli pepper were screened. Two *P. capsici* isolates, HydPak1 (MF322868) and HydPk2 (MF322869) were identified based on internal transcribed spacer (ITS) sequence homology. Plant growth promoting rhizobacteria (PGPR) play a significant role in disease suppression and plant growth promotion in various crops. Out of fifteen bacterial strains recovered from chilli rhizosphere, eight were found potential antagonists to *P. capsici *in vitro. Bacterial strains with strong antifungal potential were subjected to biochemical and molecular analysis. All tested bacterial strains, were positive for hydrogen cyanide (HCN), catalase production and indole-3-acetic acid (IAA) production (ranging from 6.10 to 56.23 µg ml^−1^), while siderophore production varied between 12.5 and 33.5%. The 16S rRNA sequence analysis of tested bacterial strains showed 98–100% identity with *Pseudomonas putida*, *P. libanensis*, *P. aeruginosa*, *Bacillus subtilis*, *B. megaterium*, and *B. cereus* sequences available in the National Center for Biotechnology Information (NCBI) GenBank nucleotide database. All sequences of identified bacteria were submitted to GenBank for accessions numbers (MH796347-50, MH796355-56, MH801129 and MH801071). Greenhouse studies concluded that all tested bacterial strains significantly suppressed the *P. capsici* infections (52.3–63%) and enhanced the plant growth characters in chilli pepper. Efficacy of many of these tested rhizobacteria is being first time reported against *P. capsici* from Pakistan. Plant growth promoting rhizobacteria (PGPR) exhibiting multiple traits may be used in the development of new, eco-friendly, and effective bioformulations as an alternative to synthetic fungicides.

## Introduction

Chilli, also red pepper or chilli pepper (*Capsicum annuum* L.) is among the extensively grown spice crop in Pakistan like many other countries around the globe. Quality and quantity of the crop are adversely affected by numerous soil-borne and areal pathogens of which *Phytophthora capsici* is one of the most devastating oomycete pathogens, resulting into damping-off and blight diseases^1^. This pathogen causes complete crop failure under favorable environmental conditions^2^. Synthetic pesticides are frequently applied to attain a high yield of the produce however, this disease management strategy comes with potential risks to the environment and human health^3^. Soil-borne nature of oomycete agents makes them more difficult to control due to longterm surviving potential in the soil^4^.

In the rhizosphere, bacteria are abundant microbes within the soil, and many of them have plant growth promotion traits known as PGPRs^5^. Their mechanism of action includes the production of indole-3-acetic acid (IAA), nitrogen fixation, soil phosphorus solubilization and various nutrients, and antagonism against pathogens by siderophores, cellulose, protease, antibiotics and cyanide production^6^. Many scientists have reported the growth promotion in cereal crops^7^, fruits^8^, vegetables^9^ including pepper^10^ by the application of rhizobacteria.

Bacterial strains belonging to Actinobacteria, *Bacillus*, *Pseudomonas*, and *Streptomyces* spp. are declared as biological control agents of *P. capsici* and suppress damping-off disease in various crops^11–13^. Because of the undesirable features of synthetic agrochemicals and increasing threat to the environment, utilization of naturally occurring biological control agents for disease suppression and plant growth enhancement is one of the possible alternatives. The present study is designed to isolate, characterize, to test the disease suppressiveness and PGP effects of native rhizobacterial strains, recovered from chilli rhizosphere. This study will help to explore the potential of these bacterial strains against other soil-borne pathogens and bio-pesticide development.

## Materials and methods

### Isolation and pathogenicity of *Phytophthora capsici*

During two years survey in chilli paper growing fields in February–November, 2016–2017, young symptomatic seedlings (15–30 days old) showing post-emergence damping-off symptoms were collected. Damping-off was confirmed by carefully observing the rotten roots^1^ and symptomatic roots showing typical browning and necrosis were collected in zip polythene bags, properly labelled, and kept in an icebox. All the collected samples were brought to the Department of Plant Pathology, PMAS Arid Agriculture University for further processing. Infected roots were surface disinfected in 0.1% sodium hypochlorite for 3 min followed by three consecutive washings in sterilized distilled water (SDW). Root tissues were cut into small slices (0.5 mm), and were placed aseptically on Petri plates containing *P. capsici* selective CMA-PARPH medium (Corn meal agar, 17 g; pimaricin, 10 mg; ampicillin, 250 mg; rifampicin, 10 mg; PCNB, 100 mg; hymexazol, 50 mg; and distilled water, 1,000 ml)^14^. All the Petri dishes were sealed with Parafilm tape, labelled with isolate code, data of isolation, and were incubated at 28 ± 2 °C for 7 days. A total of 12 *P. capsici* isolates were recovered and maintained on PDA medium amended with rifamycin (5 mg l^−1^).

Pathogenicity assay was conducted on seeds of two locally available chilli varieties (Long Green and Neelam) in vitro. Prior to seed sowing, already disinfected soil was flooded with 20 ml sporangial suspension (1 × 10^3^ sporangia ml^−1^) of *P. capsici* in 1.5 l capacity plastic pots. Seeds of both the varieties were surface sterilized and sown in infected soil (10 seeds/pot) in five repeats for each test fungal strain in a repeated experiment and un-inoculated pots were served as control. All the pots were kept 25 ± 2 °C up to 20 days. Seedling mortality percentage was observed 15 days after sowing. Re-isolation of *P. capsici* from the infected root samples confirmed the association of the pathogen with the chilli damping off disease.

### Characterization of *Phytophthora capsici*

*Phytophthora capsici* was identified based on morphological characteristics. For sporangial production, small discs (5-mm) from actively growing mycelia were cultured on V8-agar medium containing Petri plates under white fluorescent light at 26 ± 2 °C for 7 days^15^. Sterile distilled water (SDW) was added to each Petri plate, shaken to detach sporangia, and poured on a glass slide. Glass slide was covered with a coverslip and was examined under a compound microscope. Sporangial shape and size were measured at 200 × magnification while pedicle length was measured at 100 × magnification^16^ for 20 randomly chosen sporangia from each isolate. Chlamydospores production was studied in accordance with Ristaino^15^. Briefly, actively growing *P. capsici* was aseptically transferred to 25 ml of clarified V8 broth (C-V8, 163 ml clarified V8 juice, 3 g CaCO_3_, 1,000 ml distilled water, 86 mg ampicillin, 26 mg rifampicin) in a sterilized 50 ml lid containing tube, and incubated in the dark at 26 ± 2 °C for 24 h. After incubation, tubes were shaken for 5 min and were incubated in the dark for 5 days. C-V8 broth was replaced with 45 ml of SDW, followed by incubation in the dark at 18 ± 2 °C for 72 h. Chlamydospores were detached from mycelium using sterilized forceps and a dissecting needle and were observed under 100X magnification.

Two highly virulent *P. capsici* strains (HydPk1 and HydPk2) confirmed in Pathogenicity assays were subjected to molecular based identification. Genomic DNA was extracted by using the standard protocol of Cetyl Trimethylammonium Bromide (CTAB)^17,18^. The internal transcribed spacer regions (ITS1 and ITS2) of the genomic DNA were amplified by polymerase chain reaction (PCR) using universal sense ITS1 (5′-TCCGTAGGTGAACCTGCGG-3′) and ITS4 (5′-TCCTCCGCTTATTGATATGC-3′) encoding ITS-1–5.8S-ITS-2^19,20^. Reaction was carried out in 50 μl total reaction mixture volumes containing 33·25 μl grade water, 5 μl PCR (10X) buffer, 4 μl dNTPs (5 Mm each), 1·5 μl MgCl_2_ (25 mM), 2 μl each of ITS1 and IT4 primers (20 pmol), 0·25 μl (0·5 U) Taq DNA polymerase and 2 μl DNA as a template. PCR conditions were 94 °C for 5 min, followed by 30 cycles of 94 °C for 1 min, 55 °C for 1 min, and 72 °C for 2 min and a final elongation at 72 °C for 7 min. The PCR amplified products were analyzed in 2% agarose gel (high resolution agarose, Q-BIOgen) in TAE buffer containing 40 mmol l^−1^ Tris–HCl (pH 7.9), 4 mmol l^−1^ sodium acetate, and 1 mmol l^−1^ EDTA (pH 7.9). The purified PCR products were sequenced in both directions and sequences were assembled to create a final sequence for each tested isolate. Basic local alignment search tool (BLAST) analysis was performed to check the sequence similarities of the tested sequences with those already deposited sequences in the National Center for Biotechnology Information (NCBI). All the retrieved and tested sequences were aligned using ClustalW program and subjected to phylogenetic analysis. The phylogenetic tree was constructed using Neighbor-Joining (NJ) method in MEGA-X version 10.1.7 with 1,000 bootstrap replications and the evolutionary distances were calculated by using Jukes–Cantor model. Sequences were submitted to NCBI and accession numbers were obtained.

## Isolation of PGPR strains

### Soil sampling

Rhizospheric soil samples along with healthy roots were collected from the chilli pepper fields in Rawalpindi division, Pakistan (33.5651° N, 73.0169° E). Samples were labelled properly and shifted to the icebox for transportation to Plant Bacteriology Laboratory, Department of Plant Pathology, PMAS Arid Agriculture University Rawalpindi and preserved at 4 °C till further use for the isolation of rhizobacterial strains.

### Bacterial isolation and preservation

The Serial dilution procedure described by Xu and Kim^3^ was adopted for the isolation of rhizobacterial strains. One gram (1 g) of the rhizosphere soil from each sample, strongly adhering to the roots was poured separately in a test tube containing 9 ml sterile distilled water (10^−1^) and vortexed vigorously for 10 min. Subsequently, serial dilutions (from 10^−1^ to 10^−8^) were made. For the isolation of *Bacillus* spp., 100 μl of each dilution was poured on Nutrient agar (NA, Difco, USA) plates, while *Pseudomonas* spp. were isolated on Kings’ B medium incubated at 28 ± 2 °C for 24 h. Pure bacterial cultures were obtained by picking up a single discrete bacterial colony on freshly prepared NA medium plates and a total of fifteen bacterial isolates were recovered (Table 1). Obtained isolates were stored at − 80 °C in 40% glycerol and distilled water solution^21^ until further use in experiments.

**Table 1:** List of rhizobacterial strains isolated from chilli pepper fields from three different locations in Rawalpindi District, Pakistan.

S. no.	Isolates code	No. of strains	Crop	Location	Coordinates
1	AJ-RB22, AJ-RB13, JHL 3, JHL 4, JHL 8, JHL-12	6	Chilli pepper	Adiiyala Jhamra	33.4573° N, 72.9948° E
2	KSL-8T, KSL-24, RWPRB03, 4a2, RH-87, 5C	6	Chilli pepper	Kasala	15.4581° N, 36.4040° E
3	DKB53, RB09, RBT7	3	Chilli pepper	Dhok Bawa	33.4739° N, 73.0206° E

### Antagonism assay against *P. capsici *in vitro

Bacterial strains were screened in vitro for antagonism against *P. capsici*. Rhizobacterial cultures were re-streaked on NA medium 48 h before testing their antagonistic potential in dual culture^22^. A seven days old culture of HydPk2—*P. capsici* (accession MF322869) on potato dextrose agar (PDA) was used in this experiment. Briefly, 5 mm fungal mycelial plugs were cultured in the center of PDA Petri plates and bacterial cultures were streaked on both sides of the fungal plugs at a 2 cm of distance. Controls consisted of single cultures of the tested pathogen strain/s. Bacterial antagonism was tested in triplicate and plates were incubated at 28 ± 2 °C for about 96 h. The antagonistic potential was evaluated as inhibition of the mycelial radial growth of *P. capsici* against each bacterial strain tested. The experiment was carried out under a complete randomized design (CRD) with three replications.

$${\text{Mycelial}}\,{\text{inhibition}}\,\left( \% \right) = \left[ {\frac{{{\text{R}} - {\text{r}}}}{{\text{R}}}} \right] \times 100$$

where R and r is the radius of fungal mycelial growth in control and treatment, respectively.

## Biochemical features of the obtained rhizobacterial strains

### Ammonia (NH_3_) production test

The Ammonia (NH3) production test was performed in accordance with Dye^23^. In particular, the test tubes containing peptone water (10.0 g peptone; 5.0 g NaCl; 1,000 ml distilled water; 7.0 pH) were inoculated with 100 μl of 24 h grown bacterial cultures in nutrient broth medium and incubated at 28 ± 2 °C for 48–72 h. The ammonia production was detected by adding Nessler's reagent (0.5 ml) in each test tube. The production of a brown to the deep yellow colour indicated NH_3_ production.

### Hydrogen cyanide production

Hydrocyanic acid (HCN) production was evaluated according to^24^ with a modification in the procedure. In particular, 24 h bacterial cultures grown in nutrient broth medium were inoculated on TSA medium amended with 4.4 g l^−1^ glycine in Petri plates. Filter papers soaked in the picric acid solution (0.5% picric acid, 2% sodium carbonate) were put into lids of each Petri plate. These Petri plates were properly sealed with Parafilm and inoculated for 2 to 4 days at 28 ± 2 °C. After incubation for 48 h at 28 ± 2 °C, filter papers were observed for colour changes from weak yellow to reddish brown for each of the bacterial strain; indicating the positive test results.

### Catalase test

Catalase test was carried out following Hayward^25^. In particular, freshly grown (24 h old) bacterial culture on NA medium was placed on a dry glass slide and one drop of 3% H_2_O_2_ was dropped on the bacterial colony. Rapid gas bubbles formation indicated the positive test results for the tested bacterial strains.

### Potassium hydroxide (KOH) solubility test

Potassium hydroxide (KOH) solubility test was determined by following the procedure adopted by KIrsop and Doyle^26^. A loopful of 24 h old bacterial culture grown on NA medium was mixed with 3% Potassium Hydroxide solution on a dry glass slide till even suspension is formed. Formation of mucoid thread (loop) confirmed the positive reaction for the tested bacterial strains.

### IAA production assay

Indole-3-acetic acid (IAA) production in rhizobacteria was tested in accordance with Dasri, et al.^27^. Each rhizobacterial strain was suspended in distilled water at (10^8^ CFU ml^−1^) (OD620 = 0.1), then 0.5 ml aliquots were inoculated into 50 ml of King's B broth amended with 0.1% l-tryptophan followed by incubation at 28 °C for 72 h. Cultures were centrifuged at 12,000 rpm for 10 min and then 2 ml supernatant was thoroughly mixed with 4 ml of Salkowski reagent (1 ml of 0.5 M FeCl_3_ solution in 50 ml of 35% perchloric acid). Change in the colour pink to red indicated the production of IAA. Optical density (OD) was measured at 530 nm by using a spectrophotometer, and IAA concentration was determined in comparison to 10–100 µg ml^−1^ IAA standard curve. There were three replications for each bacterial strain to confirm the test results, and mean values were statistically analyzed.

### Siderophore production

Siderophore production was assessed by culturing the bacterial strains on Chrome azurol S (CAS) agar. All the tested bacterial cultures were incubated 28 ± 2 °C for 5–7 days and the siderophore production was detected by the development of a yellow-orange halo around the bacterial colonies^28^. For each strain, the siderophore production was quantified by CAS-liquid assay by mixing the bacterial culture supernatant (0.5 ml) with 0.5 ml of CAS reagent. Absorption was measured at 630 nm over a control treatment made of 0.5 ml of non-inoculated broth medium mixed with 0.5 ml of CAS reagent. There were three replications for each bacterial strain to confirm the test results, mean values were calculated, and data was statistically analyzed.

### Molecular-based identification of rhizobacterial strains

Total genomic DNA of rhizobacterial strains was isolated using GeneJet Genomic DNA purification Kit (Thermo Scientific), following the manufacturer’s instructions. Bacterial strains were identified by amplifying 16S rRNA region by PCR using a set of universal primers; 27F (5′-AGAGTTTGATCMTGGCTCAG-3) and 1492R (5′-TACGGYTACCTTGTTACGACTT-3)^29,30^. Polymerase chain reaction (PCR) reaction was carried out by using the standard reaction mixture (100 µl) containing: 5 × PCR buffer, 25 mM Mgcl_2_, 10 mM of each dNTPs, 4 µl of each primer (0.5 µM), 1 µl of Taq Polymerase enzyme (0.5 U µl^−1^), and 2 µl of bacterial DNA template. PCR conditions for 16S rRNA gene amplification were: initial denaturation of DNA template at 95 °C for 1 min per cycle, 35 cycles of denaturation at 95 °C for 15 s, annealing at 55 °C for 15 s, extension at 72 °C for 1 min and final elongation at 72 °C for 7 min. PCR amplified products were examined by separating the product on 1% agarose gel and visualized under UV transilluminator. Gel photographs were taken by using a gel documentation system. Amplified PCR product (1.5 kb) was purified using Gel and PCR Clean-Up System (Promega) and DNA quantification was carried out using NanoDrop. The amplified products were then sent to the Crop Science Department, University of Illinois Urbana-Champaign, USA for sequencing. Final sequences were obtained by joining both the reverse and forward sequences, and the 16S rRNA sequences of the bacterial strains were submitted in the GenBank database to obtain accession numbers (Table 2). The similarity between the sequences was checked by aligning test sequences, and their best-matched sequences (on average 4 to 5 sequences) available in National Center for Biotechnology Information (NCBI) GenBank nucleotide database (www.ncbi.nlm.nih.gov) using ClusterW program. The nucleotide sequence homology was also cross verified from the DNA sequences data deposited in the DNA Data Bank of Japan-DDBJ (https://www.ddbj.nig.ac.jp). Evolutionary relatedness between test sequences and retrieved sequences was determined by constructing a Maximum-Likelihood phylogenetic tree in molecular evolutionary genetics analysis (MEGA 6) software, using a Kimura 2-parameter model with Gamma distribution (K2 + G), and 1,000 bootstrap replicates.

**Table 2:** Sequence analysis based on 16S rRNA and identity with accessions available on NCBI database “16S ribosomal RNA sequences (Bacteria and Archaea).

Isolates	Identified as	Accessions	% similarity	Accessions (NCBI)
RWPRB03	*P. putida*	MH801129	99	HM486417
RBT7	*P. putida*	MH801071	99	KY982927
RB09	*P. libanensis*	MH796355	98	DQ095905
AJ-RB13	*P. aeruginosa*	MH796356	99	KF420126
DKB53	*B. subtilus*	MH796349	100	KX061099
AJ-RB22	*B. megaterium*	MH796350	100	MG544100
KSL-24	*B. cereus*	MH796347	100	KP236185
KSL-8T	*B. cereus*	MH796348	99	MF375116

### Seed germination assay

Rhizobacterial strains were tested for their effect on seed germination before proceeding pot experiments. Chilli pepper seeds (variety; Long green) were surface sterilized by dipping in 1% sodium hypochlorite for 3–5 min and washed three times in dH_2_O. Disinfected chilli seeds were soaked in 25 ml prepared concentrations of each rhizobacterial suspensions in three concentrations (10^8^ CFU ml^−1^) by gently shaking for 2 h on the shaker followed by surface drying the bacteria treated seeds on blotter paper. Ten seeds/Petri plates were placed on wet Whatman filter paper No. 41 in each Petri plate, and were incubated at 26 ± 2 °C. Filter papers were kept moist with autoclaved distilled water. Seeds soaked in autoclaved distilled water were kept as control. There were three replications for each treatment and data on seed germination percentage was recorded after 15 days of incubation. The experiment was carried out under a completely randomized design (CRD). Mean values for seed germination were calculated, and data was statistically analyzed. Seed germination percentage was calculated by the following formula:

$${\text{Seed}}\,{\text{germination}}\,\left( \% \right) = \frac{{{\text{No}}{.}\,{\text{of}}\,{\text{germinated}}\,{\text{seeds}}}}{{{\text{total}}\,{\text{no}}{.}\,{\text{of}}\,{\text{seeds}}}} \times 100.$$

### Greenhouse testing of PGPR for damping-off suppression and growth promotion

Identified rhizobacteria were subjected to check their effect on disease suppression and various plant growth parameters. Pot experiments were performed in the greenhouse located at the Department of Plant Pathology, PMAS Arid Agriculture University, Rawalpindi. Chilli (variety; Long Green) seeds were surface sterilized with 10% (v/v) sodium hypochlorite for 5 min followed by six consecutive washings in dH_2_O. Plastic pots (1.5 l) were filled with autoclaved sandy loam texture soil having physical and chemical properties as; Cation exchange capacity (18 cmol kg^−1^), pH (7.9), Organic matter (4.3 g kg^−1^), CaCO_3_ (76 g kg^−1^), Electrical conductivity, extract (0.48 dS m^−1^) Total N (0.2 g kg^−1^), Total P (267 mg kg^−1^) and Total K (198 mg kg^−1^). The soil was flooded with 20 ml sporangial suspension (1 × 10^3^ sporangia ml^−1^) of *P. capsici*. Surface sterilized seeds were dipped for 2 h in the bacterial suspension of 10^8^ CFU ml^−1^ and ten seeds per pot were sown. The control treatment consisted of pots containing soil infested with *P. capsici* inoculum and distilled water-treated chilli seeds (without bacterial inoculation). All the pots were arranged in complete randomized designed (CRD) in five replications and were placed in the greenhouse. Disease severity was taken on the disease rating scale (DRS) representing 0 = healthy plant; 1 = 1 to 30% wilting; 2 = 31 to 50% wilting; 3 = 51 to 70% wilting; 4 = 71 to 90% wilting; 5 = more than 90% wilting or dead plant^31^. Data on damping-off disease suppression was recorded 15 days after seed sowing while plant growth parameters were recorded 30 days after sowing.

#### Statistical analysis

Statistical analysis was performed using Statistix 8.1 software and Microsoft Office Excel 2010. A completely randomized design (CRD) was used for all experiments with replicated treatments. Mean values for each treatment were calculated, and all the treatment means were compared via Analysis of variance (ANOVA) using the least significant differences test (LSD) at 5% (*P* ≤ 0.05) probability level.

## Results

### *Phytophthora capsici* and pathogenicity assay

**Figure 1:**
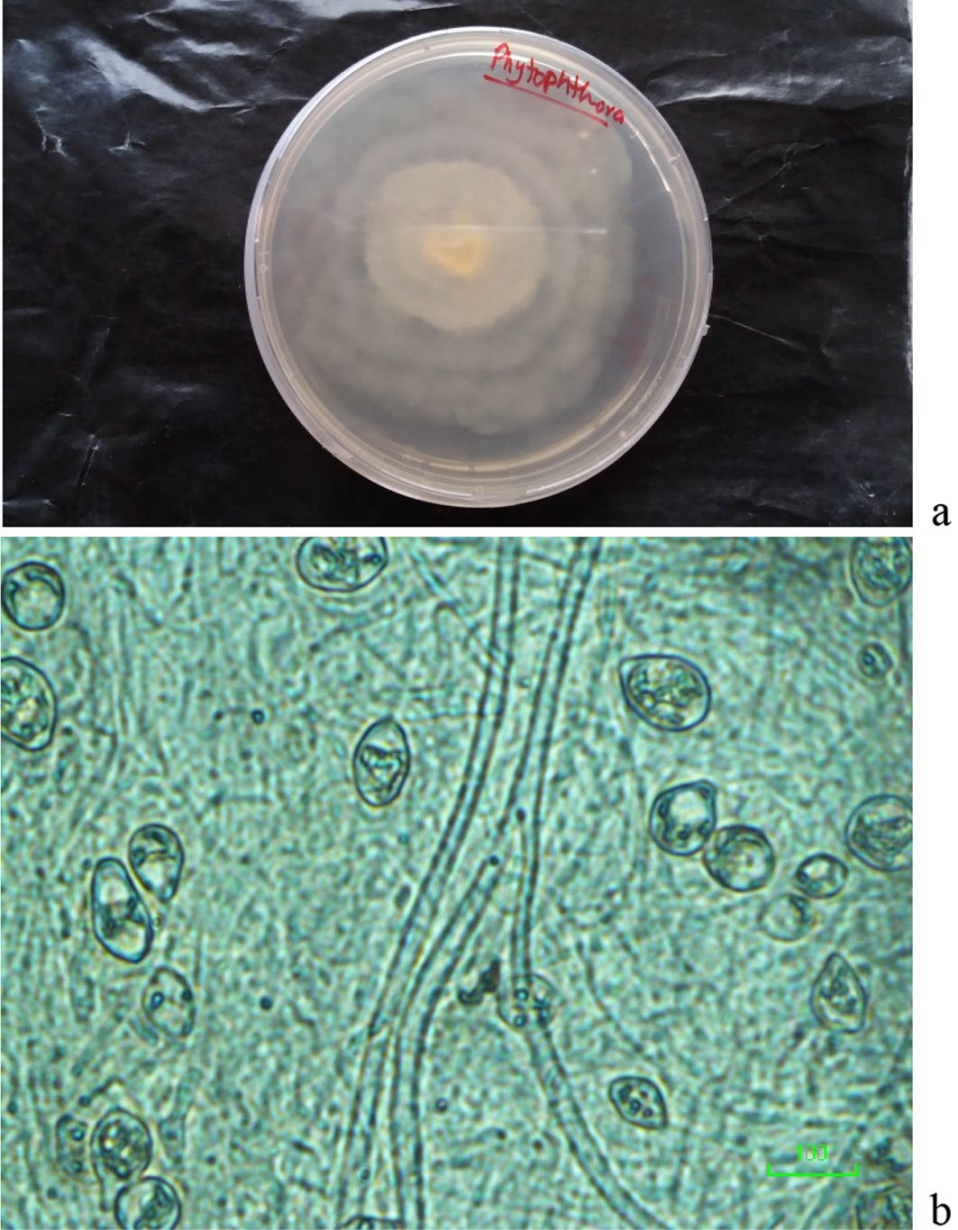
(**a**) Pure culture of *P. capsici* on CMA-PARPH medium, (**b**) microscopic image of *P. capsici*.

**Figure 2:**
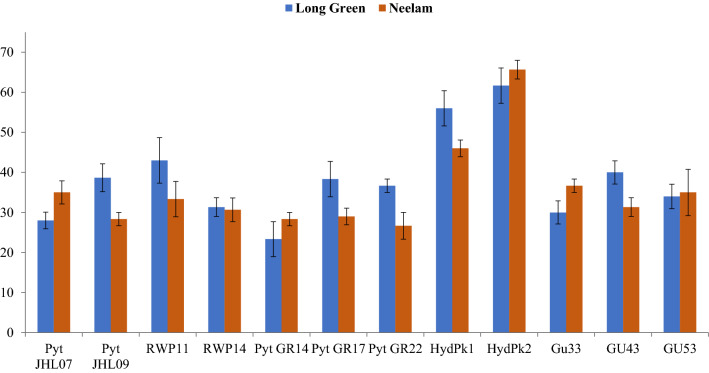
Pathogenicity assay to test seedling mortality percentage in chilli pepper by *P. capsici*. Long Green and Neelam are the two chilli pepper varieties. Pathogenicity assay was carried out in five replications for each treatment and data on seedling mortality percentage was recorded 15 days after treatment. Mean values were calculated and statistical analysis was performed using Statistix 8.1. All the mean values were subjected to analysis of variance, and means were separated by LSD test at 5% probability. Error bars represent the standard error values of the means.

A total of twelve *P. capsici* isolates were recovered from infected chilli pepper root samples, collected during a survey in Rawalpindi region (33.5651° N, 73.0169° E). Pure culture of *P. capsici* and the microscopic images are shown (Fig. 1). Two separate experiments under the same environmental conditions were conducted to check the pathogen virulence on the chilli pepper. All the tested isolates varied in their virulence to chilli seedlings and mortality percentage was recorded which ranged from 23.3 to 61.7% on *cv.* Long Green and 26.7–65.7% on *cv.* Neelam ( Fig. 2). Out of 12 tested isolates, HydPk1 and HydPk2 showed the highest percentage seedling mortality in both the chilli varieties were ranged 46–56% and 62–66% respectively (Fig. 2) and were highly virulent. The isolate HydPk2 showed maximum seedling mortality on both cultivars; 62% and 66%, it was selected to test in management trials in vitro and in greenhouse.

### Characterization of *Phytophthora capsici*

**Figure 3:**
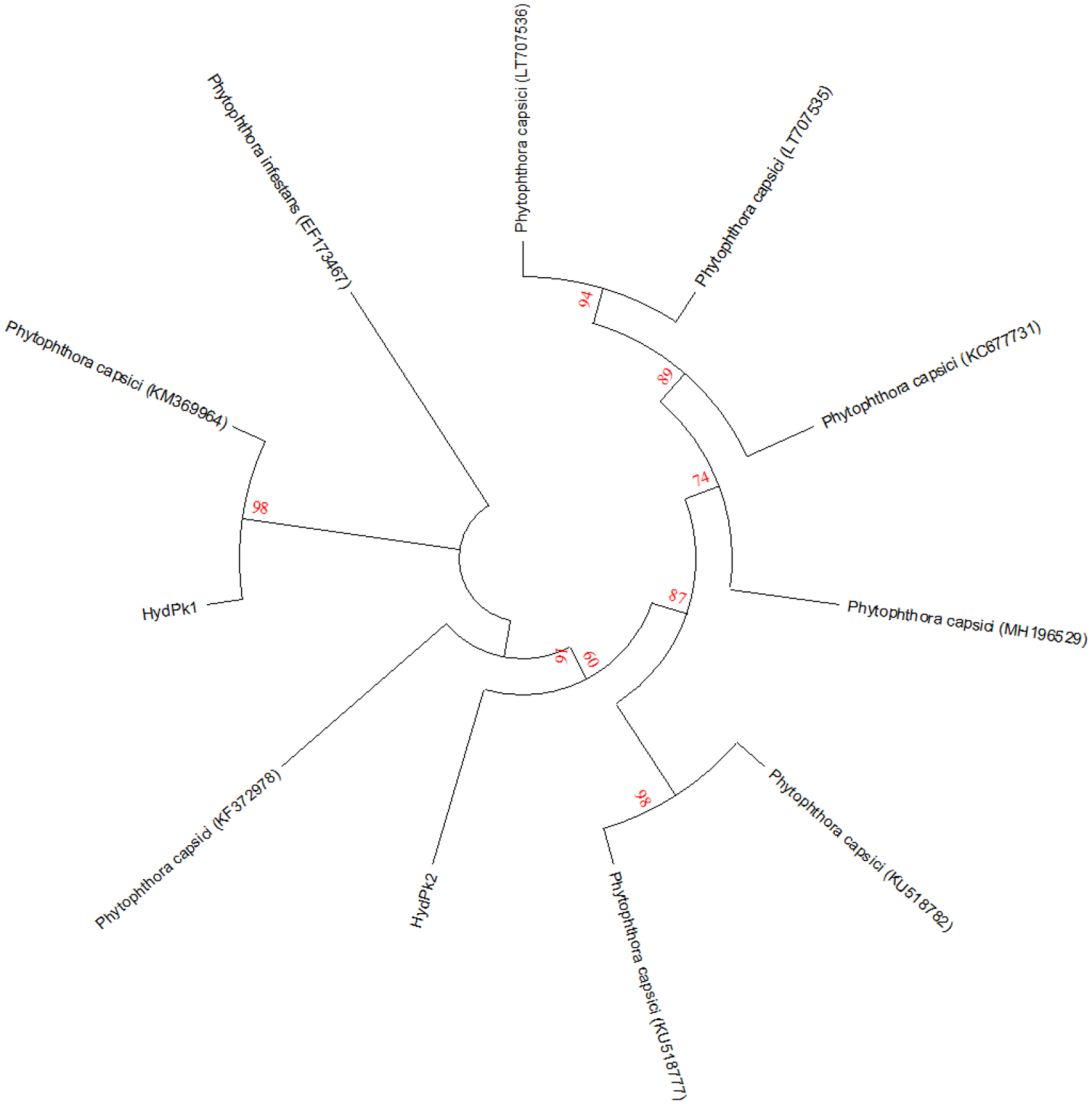
Phylogenetic relationship between the identified strains and representative *P. capsici* sequences. ITS1 and ITS2 regions of the tested *P. capsici* isolates were amplified, and all the retrieved and tested sequences were aligned using the CLUSTAL W program. The phylogenetic tree was constructed using Neighbor-Joining (NJ) method in MEGA-X version 10.1.7 with 1,000 bootstrap replications and the evolutionary distances were calculated by using Jukes–Cantor model.

All the isolates produced ovoid, papillate sporangia except RWP14 and GU43 which produced spherical, papillated sporangia. The mean sporangial length among the isolates ranged from 44.7 to 53.1 µm while the sporangial width varied from 24 to 38.4 µm. All the isolates showed pedicle length ranged from 30.6 to 75.5 µm. Maximum pedicle length of 75.5 µm was shown by RWP11 while minimum pedicle length 30.6 µm was observed in HydPk1. Chlamydospores diameter was also recorded in the range of 20.7–29.7 µm (Table 3). Two highly virulent *P. capsici* (HydPk1 and HydPk2) were subjected to molecular characterization. Internal transcribed spacer regions ITS1 and ITS2 were amplified in both forward and reverse directions and BLAST analysis confirmed 99% identity with those already deposited ITS sequences of *P. capsici* (KM369964 (Mexico) and KU518782 (India). Evolutionary relationship of all the sequence was determined by constructing a phylogenetic tree (Fig. 3). Sequences of HydPk1 and HydPk2 were submitted to GenBank nucleotide database and accessions MF322868 and MF322869 were obtained.

**Table 3:** Morphological features of isolates of *Phytophthora capsici* isolated from chilli pepper fields.

Isolate code	Sporangial shape	Sporangial length (µm)	Sporangial width (µm)	Pedicle length (µm)	Chlamydospores diameter (µm)
Pyt JHL07	Ovoid-papillate	45.8 ± 3.1^ab^	35.7 ± 6.3^abc^	71.9 ± 4.4^ab^	20.7 ± 1.1^b^
Pyt JHL09	Ovoid-papillate	50.0 ± 2.5^ab^	32.0 ± 3.7^abcd^	70.8 ± 4.2^ab^	29.7 ± 1.7^a^
RWP11	Ovoid-papillate	52.1 ± 2.3^ab^	36.6 ± 4.9^ab^	75.5 ± 4.7^a^	24.3 ± 2.9^ab^
RWP14	Spherical-papillate	53.1 ± 4.4^a^	24.0 ± 1.3^d^	65.8 ± 2.5^ab^	29.0 ± 1.6^a^
Pyt GR14	Ovoid-papillate	46.4 ± 1.8^ab^	38.4 ± 3.2^a^	39.6 ± 3.1^cd^	24.8 ± 2.7^ab^
Pyt GR17	Ovoid-papillate	45.8 ± 2.8^ab^	34.2 ± 3.4^abcd^	69.9 ± 5.7^ab^	23.6 ± 2.0^ab^
Pyt GR22	Ovoid-papillate	49.0 ± 2.5^ab^	29.8 ± 2.6^abcd^	61.2 ± 3.3^b^	25.3 ± 3.5^ab^
HydPk1	Ovoid-papillate	45.8 ± 2.2^ab^	27.3 ± 2.9^bcd^	30.6 ± 2.1^d^	24.7 ± 1.0^ab^
HydPk2	Ovoid-papillate	44.7 ± 2.2^b^	26.1 ± 2.5^cd^	37.8 ± 4.5^cd^	25.3 ± 1.4^ab^
Gu33	Ovoid-papillate	50.6 ± 3.0^ab^	29.7 ± 2.0^abcd^	74.9 ± 3.8^a^	24.2 ± 2.1^ab^
GU43	Spherical-papillate	49.1 ± 4.0^ab^	35.6 ± 4.1^abc^	42.5 ± 3.6^c^	26.3 ± 1.6^ab^
GU53	Ovoid-papillate	47.3 ± 2.6 ^ab^	35.1 ± 3.9^abc^	41.0 ± 3.3^cd^	27.9 ± 2.2^ab^
LSD		8.1734	10.310	11.097	6.0214

All the presented values are means of twenty replicates. All the means were subjected to analysis of variance and means were separated by LSD test. Letters represent the significant difference among the mean values and ± are standard error values of the means.

### Antagonism assay against *P. capsici *in vitro

Antagonism assay was performed to check the potential of rhizobacterial strains in mycelial growth inhibition against *P. capsici *in vitro. All the tested bacterial strains showed significant antagonism activity against *P. capsici* in dual culture assay on PDA (Table 4). Out of 15 tested agents, eight bacterial strains were found potential agents against *P. capsici* and significantly inhibited the mycelial growth 96 h after incubation. Fungal growth inhibition (cm) was ranged 61.7–88.3% over un-inoculated control. Maximum fungal mycelial growth inhibition was done by KSL-8T—*Bacillus cereus* (88.3%), AJ-RB13—*Pseudomonas aeruginosa* (85.7%) and RWPRB03—*Pseudomonas putida* (81.1%) while AJ-RB22—*Bacillus megatarium* was least effective (61.7%) among all the tested bacterial agents over untreated control.

**Table 4:** In vitro antifungal activity of rhizobacteria strains on *Phytophthora capsici*.

S. no.	Bacterial strains	Fungal mycelia growth 96 h after incubation	Percentage inhibition
1	RWPRB03—*Pseudomonas putida*	0.9 ± 0.08^de^	81.1
2	RBT7—*Pseudomonas putida*	1.3 ± 0.08^cd^	74
3	RB09—*Pseudomonas libansis*	1.6 ± 0.19^bc^	68.2
4	AJ-RB13—*Pseudomonas aeruginosa*	0.7 ± 0.08^e^	85.7
5	DKB53—*Bacilus subtilus*	1.6 ± 0.21^bc^	68.8
6	AJ-RB22—*Bacilus megatarium*	1.97 ± 0.12^b^	61.7
7	KSL-24—*Bacilus cereus*	1.3 ± 0.18^cd^	75.3
8	KSL-8T—*Bacilus cereus*	0.6 ± 0.15^e^	88.3
9	Control	5.1 ± 0.12^a^	0
LSD value		0.4253	

All the presented values are means of three replicates. Means were subjected to analysis of variance and were separated by LSD test. Letters represent the significant difference among the mean values and ± are standard error values of the means.

### Biochemical analysis

Bacterial strains with promising antifungal potential; RWPRB03—*Pseudomonas putida*, RBT7—*Pseudomonas putida,* RB09—*Pseudomonas libansis*, AJ-RB13—*Pseudomonas aeruginosa*, DKB53—*Bacillus subtilus*, AJ-RB22—*Bacillus megatarium*, KSL-24—*Bacillus cereus* and KSL-8T—*Bacillus cereus* were subjected to biochemical analysis and plant growth promoting (PGP) traits. All the rhizobacteria produced ammonia except strain AJ-RB13, while HCN and catalase test results were positive for all the bacterial strains. For Potassium hydroxide (KOH) test, bacteria belonging to *Pseudomonas* spp. showed positive response while bacteria from *Bacillus* spp. showed a negative test results. All the bacterial strains significantly produced IAA, and IAA production was quantified ranging (6.1–56.2 µg ml^−1^). Maximum IAA was produced by KSL-24—*Bacillus cereus* (56.2 ± 2.58 µg ml^−1^) followed by RWPRB03—*Pseudomonas putida* (35.9 µg ml^−1^) and KSL-8T—*Bacillus cereus* (29.7 µg ml^−1^). Siderophore production was exhibited by all the tested rhizobacterial strains ranging (12.5—33.5%). Highest siderophore production percentage was produced by RWPRB03—*Pseudomonas putida* (33.5%) followed by AJ-RB13—*Pseudomonas aeruginosa* (31.6%) and AJ-RB22—*Bacillus megatarium* (29.6%) while this activity was observed least in KSL-8T—*Bacillus cereus* (12.5%) and the results are presented in (Table 5).

**Table 5:** Biochemical and plant growth promoting traits of the tested bacterial isolated from chilli pepper.

Rhizobacterial isolate	AP	HCP	CT	KT	IAA production (µg ml^−1^)		Siderophore production (%)
					Without tryptophan	With tryptophan	
RWPRB03—*Pseudomonas putida*	+	+	+	+	5.6 ± 0.52^a^	35.9 ± 3.10^b^	33.5 ± 0.9^a^
RBT7—*P. putida*	+	+	+	+	2.4 ± 0.09^de^	16.9 ± 3.10^e^	26.8 ± 1.7^c^
RB09—*P. libansis*	+	+	+	+	3.6 ± 0.60^bcd^	19.9 ± 1.65^de^	21.2 ± 1.4^d^
AJ-RB13—*P. aeruginosa*	−	+	+	+	2.9 ± 0.54^cd^	6.1 ± 1.03^f^	31.6 ± 1.6^ab^
DKB53—*Bacilus subtilus*	+	+	+	−	5.4 ± 1.27^a^	17.3 ± 1.10^e^	16.7 ± 1.7^e^
AJ-RB22—*B. megatarium*	±	+	+	−	1.6 ± 0.52^e^	22.9 ± 3.19^d^	29.6 ± 0.8^b^
KSL-24—*B. cereus*	+	+	+	−	3.7 ± 0.60^bc^	56.2 ± 2.58^a^	16.4 ± 1.0^e^
KSL-8T—*B. cereus*	+	+	+	−	4.2 ± 0.65^b^	29.7 ± 3.52^c^	12.5 ± 0.3^f^
LSD value					1.1588	4.4717	2.1883

All the presented values are means of three replicates. Means were subjected to analysis of variance and were separated by LSD test. Letters represent the significant difference among the mean values and ± are standard error values of the means.

*AP* ammonia production, *HCP* hydrogen cyanide production, *CT* catalase test, *KT* KOH test.

### Molecular-based identification of rhizobacterial strains

**Figure 4:**
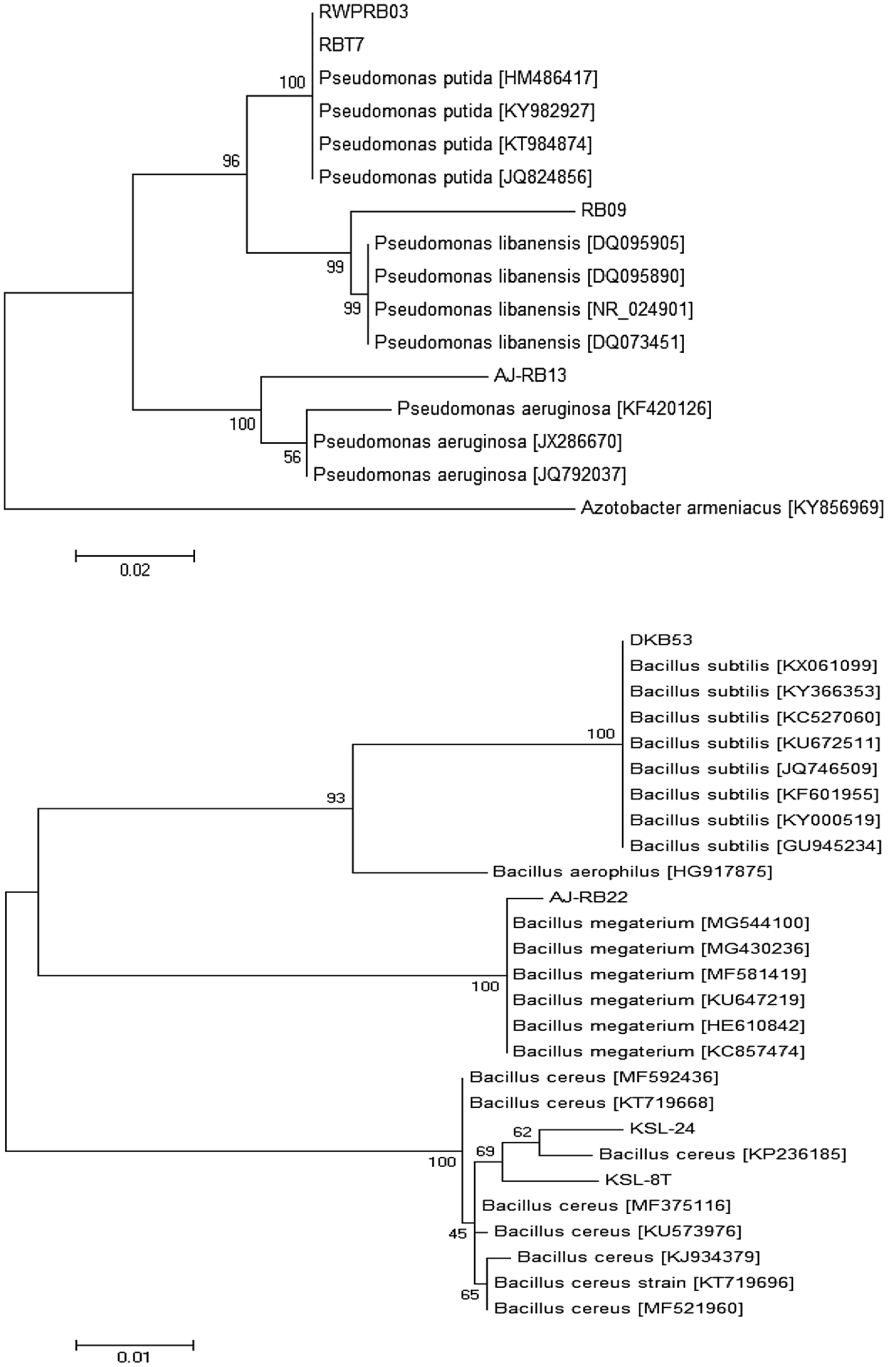
Phylogenetic relationship between the identified *Bacillus* and *Pseudomonas* strains and representative bacterial species based on 16S rRNA gene sequences developed with the ClustarW program in MEGA6 and constructed using Maximum-Likelihood method with 1,000 bootstrap replicates. The values indicate the percentage of clustering matches. Sequence closest matches were based on the NCBI database “16S ribosomal RNA sequences. The scale bar indicates the number of differences in base composition among sequences.

Bacterial strains with promising antagonistic potential and biochemical traits were identified based on 16S rRNA sequence analysis. Phylogenetic trees constructed from 16S rRNA sequences showed that tested bacterial strains were belonging to *Pseudomonas* and *Bacillus* genus (Fig. 4). The Maximum-Likelihood tree indicated that RWPRB03 and RBT7 clustered with *P. putida*, and showed 99% identity with accessions; HM486417 and KY982927. Bacterial strain RB09 was 98% identical to *P. libanensis* (DQ095905), while AJ-RB13 clustered with *P. aeruginosa* and showed 99% sequence homology with accession KF420126. The other four strains viz., DKB53, AJ-RB22, KSL-24 and KSL-8T were closely related to *B. subtilus*, *B. megatarium* and *B. cereus* and showed 99 to 100% identity with accessions: KX061099, MG544100, KP236185 and MF375116 respectively (Table 2).

### Seed germination assay

Seed germination assay was performed to investigate any positive or negative impact of bacterial strains on chilli seeds at germination stage. The effect of bacterial seed treatment upon seed germination varied with different strains. All bacterial treatments showed a significant effect on the seed germination percentage as compared to control. Seed germination percentage (%) was observed ranging from 73.3 to 93.3% in all the treatments and no stress on seed germination was observed in any treatment. Maximum seed germination was observed in chilli seeds dressed with *P. putida* (93.3%) followed by *P. libanensis* and *B. subtilus* (86.7%). Germination was observed slightly low (73.3%) in *P. aeruginosa* treated seeds among all the treatments compared to untreated control (Table 6). These results showed that rhizobacterial seed treatment could improve the chilli seed germination without posing any negative impact.

**Table 6:** Effect of antagonistic bacterial strains on chilli pepper seed germination in vitro.

Bacterial isolates (10^8^ cfu ml^−1^)	Chilli pepper seed germination (%)
RWPRB03—*Pseudomonas putida*	93.3 ± 5.77a
RBT7—*Pseudomonas putida*	83.3 ± 5.77abc
RB09—*Pseudomonas libanensis*	86.7 ± 5.77ab
AJ-RB13—*Pseudomonas aeruginosa*	73.3 ± 5.77c
DKB53—*Bacillus subtilis*	86.7 ± 5.77ab
AJ-RB22—*Bacillus megatarium*	80.0 ± 10.0bc
KSL-24—*Bacillus cereus*	83.3 ± 11.6abc
KSL-8T—*Bacillus cereus*	76.7 ± 5.77bc
Control	86.7 ± 5.77ab
LSD	12.352

All the presented values are means of three replicates. Means were subjected to analysis of variance and were separated by LSD test. Letters represent the significant difference among the mean values and ± are standard error values of the means.

### Greenhouse testing of PGPR for damping-off suppression and PGP effect

Rhizobacteria with high antifungal potential were evaluated for disease suppression and plant growth promotion traits in pot trials under greenhouse conditions. All the tested bacterial strains significantly enhanced the seed germination ranged (75.6–91.1%) as compared to control treatment (57.8%) and reduced the seed mortality caused by *P. capsici*. However, maximum seed germination was done by DKB53—*Bacillus subtilus* (91.1%) followed by AJ-RB22—*Bacillus megatarium* (88.9%) and RWPRB03—*Pseudomonas putida* and KSL-8T—*Bacillus cereus* (86.7%). Plant growth characters viz., shoot and root length (cm), fresh and dry shoot and root weight (g) were significantly enhanced by the bacterial seed inoculation as compared to untreated control. All the rhizobacterial strains enhanced the shoot and root length in range (9.47–14.67 and 3.30–5.63 in cm) as compared to control where shoot and root length was 3.43 cm and 1.07 cm respectively. All the tested bacterial strains showed a strong ability to produce IAA, thus aided to enhance plant growth characters in chilli pepper. An increase in fresh shoot and root weight was also observed against untreated control treatment. Dry shoot and root weight were also significantly increased over untreated control (Table 7).

**Table 7:** Effect of bacterial isolates on disease suppressiveness and plant growth promotion in chilli pepper in greenhouse conditions.

Rhizobacterial strains	GP	DRS	MP	Plant growth parameters					
				SL (cm)	RL (cm)	FSW (g)	FRW (g)	DSW (g)	DRW (g)
RWPRB03—*Pseudomonas putida*	86.7 ± 3.8^ab^	1	13.3 ± 3.8^bc^	11.47 ± 0.58^cd^	4.67 ± 0.27^bc^	2.3 ± 0.17^a^	1.8 ± 0.19^ab^	0.51 ± 0.03^d^	0.42 ± 0.02^c^
RBT7—*P. putida*	80.0 ± 3.8^ab^	1	20 ± 3.8^bc^	10.57 ± 0.52^de^	4.27 ± 0.15^bc^	2.1 ± 0.15^ab^	1.3 ± 0.20^bcd^	0.66 ± 0.05^c^	0.51 ± 0.04^bc^
RB09—*P. libanensis*	84.4 ± 5.9^ab^	1	15.6 ± 5.9^bc^	13.97 ± 0.35^ab^	5.03 ± 0.44^ab^	1.5 ± 0.23^c^	1.6 ± 0.17^abcd^	1.08 ± 0.03^a^	0.73 ± 0.03^a^
AJ-RB13—*P. aeruginosa*	75.6 ± 5.9^b^	1	24.4 ± 5.9^b^	11.37 ± 0.66^cd^	3.30 ± 0.26^d^	2.3 ± 0.22^a^	1.2 ± 0.18^d^	0.92 ± 0.11^b^	0.44 ± 0.03^bc^
DKB53—*Bacillus subtilus*	91.1 ± 2.2^a^	1	8.9 ± 2.2^c^	9.47 ± 0.75^e^	4.30 ± 0.21^bc^	2.0 ± 0.21^abc^	1.7 ± 0.18^abc^	0.61 ± 0.03^cd^	0.42 ± 0.04^c^
AJ-RB22—*B. megatarium*	88.9 ± 4.4^a^	1	11.1 ± 4.4^c^	14.67 ± 0.66^a^	5.63 ± 0.20^a^	1.8 ± 0.19^abc^	1.8 ± 0.09^a^	1.13 ± 0.05^a^	0.78 ± 0.03^a^
KSL-24—*B. cereus*	80.0 ± 3.8^ab^	1	20 ± 3.8^bc^	9.60 ± 0.32^e^	4.03 ± 0.38^cd^	2.2 ± 0.20^ab^	1.3 ± 0.09^cd^	0.72 ± 0.05^c^	0.47 ± 0.03^bc^
KSL-8T—*B. cereus*	86.7 ± 3.8^ab^	1	13.3 ± 3.8^bc^	12.5 ± 0.51^bc^	4.43 ± 0.41^bc^	1.6 ± 0.18^bc^	1.2 ± 0.21^cd^	0.59 ± 0.03^cd^	0.52 ± 0.03^b^
Control (untreated)	57.8 ± 2.2^c^	3	42.2 ± 2.2^a^	3.43 ± 0.45^f^	1.07 ± 0.30^e^	0.8 ± 0.19^d^	0.5 ± 0.12^e^	0.29 ± 0.03^e^	0.2 ± 0.02^d^
LSD value	12.454		12.454	1.6417	0.9119	0.5756	0.4863	0.1485	0.0902

All the presented values are means of five replicates. Means were subjected to analysis of variance, and means were separated by LSD test. Letters represent the significant difference among the mean values and ± are standard error values of the means. Germination percentage and disease data was recorded 15 days after treatment while data on growth promotion parameters was recorded 30 days after sowing. All the results were compared with untreated control where only *P. capsici* was applied with no bacterial seed inoculation.

*GP* germination percentage, *DRS* disease rating scale, *MP* mortality percentage, *SL* shoot length, *RL* root length, *FSW* fresh shoot weight, *FRW* fresh root weight, *DSW* dry shoot weight, *DRW* dry root weight.

## Discussion

*Phytophthora capsici* is the most destructive plant pathogen, that poses a serious threat by infecting the host plants at any growth stage and causes seedling death, crown rot, foliar blight, and fruit rot. The pathogen causes severe losses in many crops like cucurbits, eggplant, pepper, and tomato^15,32^. *Phytophthora capsici* is soil borne in nature and can survive for a long time in soil by forming oospores. Availability of free water support asexual reproduction and pathogen form sporangia and zoospores^33^ which are dispersed with soil, water, and air currents^34^. In the present study, *P. capsici* was isolated from damping-off chilli root samples showing the characteristic symptoms of rotten roots, decay, wilt and necrosis^35^ and a total of 12 isolates were recovered and purified. Pathogenicity assay was performed to screen the most virulent isolates as different isolates have varied level of virulence. A research study suggested that *P. capsici* isolates from pepper and pumpkin differ in virulence levels^32^. Morphologically identification confirmed the production of papillate sporangia on long pedicels, and Chlamydospores and these findings are supported by a study of^36^. According to^37^, Chamydospores production is not much common in *Capsicum* isolates but the formation of Chamydospores in *P. capsici* isolates depends on the cultural methods and the origin of the host^38^. Two most aggressive isolates (HydPk1 and HydPak2) screened in pathogenicity assays were subjected to molecular identification by amplifying ITS1 and ITS2 regions^19,20^, and were identified as *P. capsici*. In another study, pathogen was identified as *Phytophthora colocasiae* on the basis of internal transcribed spacer (ITS) sequence homology^39^. Other researchers have also identified *P. capsici* by amplifying ITS regions of the isolates collected from chilli pepper blight samples^40^.

Rhizobacteria colonizing the rhizosphere interact with crops in various ways; by controlling the plant diseases by antagonism and by promoting plant growth parameters^41^. The interaction of beneficial rhizobacteria and plant-phytopathogen could offer new strategies to enhance plant productivity in an environment friendly way^42^. Rhizobacteria with plant growth promoting potential are used as an alternative to chemical pesticides in the agriculture industry^43^. Various researches have proved that upon attack by soil-borne fungal pathogens, plants can exploit microbial consortia from the soil for protection against infections, restructuring bacterial communities associated with rhizosphere^44^. In our study, fifteen rhizobacterial strains were recovered from chilli rhizosphere, majorly belonged to the genera *Bacillus* and *Pseudomonas*. Our findings are comparable to various reports on biocontrol potential of bacteria belonging to genera *Achromobacter*, *Arthrobacter*, *Azospirillum*, *Azotobacter*, *Bacillus*, *Clostridium*, *Enterobacter*, *Flavobacterium*, *Micrococcus* and *Pseudomonas* being the most common bacterial groups prevailing in soil^45,46^.

Biological control of *P. capsici* can be due to antagonistic ability of the bacterial strains^47,48^. Initially, fifteen bacterial strains were screened for antagonism against *P. capsici*. All the tested bacterial strains showed varied levels of antagonistic potential. Results revealed that five bacterial isolates viz., RWPRB03—*Pseudomonas putida*, RBT7—*Pseudomonas putida*, AJ-RB13—*Pseudomonas aeruginosa*, KSL-24—*Bacillus cereus*, and KSL-8T—*Bacillus cereus* showed > 70% mycelial growth inhibition of *P. capsici*. In a research study, out of 811 tested rhizospheric bacteria, five bacterial strains showed highest antagonistic potential against three most prevalent strains of bacterial leaf blight (BLB) pathogen *Xanthomonas oryzae* pv. *oryzae*^49^. Biocontrol potential of tested bacterial isolates could be due to antibiosis and various antibiotics have been previously identified and reported by many researchers^50,51^. It has been reported that production of various antimicrobial compounds are responsible for fungal growth inhibition as these compounds result into cytolysis, leakage of potassium ions, disruption of the structural integrity of membranes, inhibition of mycelial growth, spore germination inhibition and protein biosynthesis^52^. Antifungal potential of *Bacillus* spp. attributed to the production of various levels of different lipopeptides against varying fungal phytopathogens^53^ while Dimethyl disulfide and other sulfur-containing compounds production by *Pseudomonas* spp. were studied to stop the mycelial growth of *P. infestans*^54^ and various other volatile compounds emitted by *Pseudomonas* spp., such as 1-Undecene, also reduce sporangia formation and release of zoospores in *P. infestans*^55^.

Most of the tested bacteria exhibited multiple PGP traits which aid to growth promotion and disease reduction capability of PGPR. Such multiple modes of action have been researched to be the prime reasons for the plant growth promotion and disease suppressing potential of PGPR^56^. It is important to explore the potential of native rhizobacterial strains for their PGP traits to get the desired benefits on disease control and plant growth promotions. In this study, eight bacterial strains with strong antagonistic potential were screened for biochemical and plant growth promotion characters. All tested bacterial strains showed a positive response to ammonia production, Hydrogen cyanide (HCN) production, catalase reaction, KOH test (*Bacillus* spp.), IAA and siderophore production. Ammonia (NH_3_) production is important feature of PGPR, which indirectly enhances the plant growth^57^. It accumulates and supply nitrogen to their host plants and promotes plant growth^58^. Various studies have reported the ammonia production by rhizobacteria^3,59,60^. In our studies, except one bacterial strain, all were positive for ammonia production. All the tested bacterial strains were positive for HCN production test. It was originally believed that HCN production play its role in plant growth promotion by suppressing the plant pathogens^61,62^. However, this concept has recently been changed. It has been believed that HCN production indirectly increases phosphorus availability by chelation and sequestration of metals, and indirectly increases the nutrient availability to the rhizobacteria and host plants^63^. The production of HCN by PGPR are independent of their genus, thus they are used as biofertilizers or biocontrol to enhance crop production^64^ and these are being used as biofertilizer in growth promotion and yield enhancement. All the tested bacterial strains were catalase positive. Previous studies have reported the production of antioxidant enzymes like catalase by rhizobacteria^65^ which suppressed early blight disease in tomato^66^ and induced resistance against tomato yellow leaf curl virus^67^. Indole-3-acetic acid (IAA) is secondary metabolites, and its production in bacterial agents is generally described based on their ability to use tryptophan supplemented in the growth medium, which is the major precursor of IAA biosynthesis via indole pyruvic acid (IPA) pathway^68^. It supports root development, elongation and proliferation and help plants to take up water and nutrients from soil^69^. All the tested bacterial strains produced IAA in various concentrations and this varying ability could be due to the difference in bacterial physiological characters, however^70^ reported that Indolepyruvic decarboxylase (IPDC) is the enzyme which determines IAA biosynthesis. All the tested strains belonging to *Bacillus* and *Pseudomonas* spp. produced IAA even in the growth medium without tryptophan as supplementary material. Siderophore was also produced by all the tested bacterial strains ranged from 12.5 to 33.5%. These findings are further supported by previous reports in which siderophore production was reported as an important mechanism involved in the suppression of bacterial leaf blight (BLB) disease^71^. Siderophore is one of the major biocontrol mechanisms exhibited by various plant growth promoting rhizobacterial groups under iron-limiting condition. PGPR produces a wide range of siderophore which has a high affinity for iron thus, lowering the availability of iron to pathogenic agents including plant pathogenic fungi^72^. Siderophore production by PGPR controls soil-borne pathogenic fungi by limiting iron availability to them^73^. The antagonistic potential of the selected bacterial strains might be due to the siderophores and HCN production or synergistic interaction of these two or with other metabolites^49^.

The 16S rRNA sequences have been widely used in the classification and identification of Bacteria and Archaea^74^. In our studies, 16S rRNA sequence based maximum-likelihood tree indicated that tested bacterial strains RWPRB03 and RBT7 showed 99% sequence homology with *P. putida* (accessions; HM486417 and KY982927), while RB09 was 98% identical to *P. libanensis* (DQ095905). AJ-RB13 clustered with *P. aeruginosa* and showed 99% homology with accession KF420126. The other four bacterial strains viz., DKB53, AJ-RB22, KSL-24 and KSL-8T showed 99 to 100% sequence homology with accessions: KX061099, MG544100, KP236185 and MF375116 respectively. Similarly, Ref^75^ reported that 16S rRNA sequence based characterization indicated that most of the bacterial strains isolated from cucumber rhizosphere belonged to *Pseudomonas stutzeri*, *Bacillus subtilis*, *Stenotrophomonas maltophilia*, and *B. amyloliquefaciens*.

Studies have proved that seed treatment is an effective strategy to enhance seedling emergence, seed vigor, and to prevent seed and soil borne pathogens^76^. Chemical seed treatment is a major practice being followed to prevent damping off disease^77,78^. Many kinds of chemicals are used to remove the pathogen inoculum from seed coats^76^ but these chemicals could negatively affect seed germination, cause phytotoxicity^79^, pose a negative impact on human health and environment^80^. Results from our studies indicated that seed treatment with bacterial strains significantly enhanced the seed germination over untreated control, and no phytotoxicity was observed in any treatment and our results are supported by the finding of^75^. In a study, high amylase activity during germination was observed in rice and legume inoculated with PGPR^79^ which support the root and shoot germination in young seedlings.

Results from our study indicate that seed treatment with PGPR significantly reduced the seedling mortality and disease severity of *P. capsici* in chilli pepper and studies have proved the role of rhizobacteria as biological control agents^81,82^. Various studies have reported the antifungal potential of rhizobacteria belonging to *Pseudomonas* and *Bacillus* spp. against *P. infestans* and *P. capsici*^83,84^. Results from our study showed that eight bacterial strains significantly suppress the damping off disease, and enhanced the plant growth in pot experiments. The results from greenhouse trials suggested that the tested PGPR slightly differ in their effects on disease suppression and PGP traits in chilli, and all the tested bacterial strains performed better under greenhouse conditions compared to untreated control. Earlier researches have revealed that the PGP effects and disease reduction potential of rhizobacteria are attributed to multiple traits^56^. Our results on chilli disease suppression and PGP are supported by other studies on growth promotion by PGPR in common bean^85^ and tomato^86^. In another study, strains of PGPR from cucumber plants rhizosphere were tested for their plant growth promoting traits and antifungal potential against Phytophthora crown rot of cucumber seedlings under in vitro and greenhouse conditions. Results revealed that tested rhizobacterial strains protected the plants by various modes of action including antibiosis against target pathogen, competitive colonization, and plant growth promotion^75^. These findings support the utilization of these rhizobacterial strains for bioformulations development their commercial use as biocontrol agents in the open fields.

It has been reported that the field application of bacterial based products has been hampered because of their low performance due to various climatic and soil factors^87,88^. This is important to test the efficacy of native rhizobacteria under the same environmental condition where they would be used in plant growth promotion. It was concluded in various studies that environmental factors significantly influence the rhizobacterial colonization, biological activates, and disease suppressing potential^89–91^. In the majority of the cases, bacterial based formulations imported from the other counters bearing different climatic conditions failed to perform up to their maximum potential under warm environmental conditions prevailing in Pakistan. In this study, bacterial strains were isolated from the local fields located in Pakistan and were tested for their potential to suppress the soil-borne oomycetes and plant growth promotion in chilli pepper. Development of bioformulations on available carrier materials is undergoing. To find out optimized formulations dose level and appropriate application method, it requires a series of experiments with different soil types under greenhouse and open field conditions, and these studies are under progress.

## Conclusions

Out of fifteen, eight bacterial strains efficiently suppressed the mycelial growth of pathogenic *Phytophthora capsici* in direct interactions-assays in vitro. Bacterial strains with strong anti-fungal potential were found positive for HCN, catalase test, Indole-3-acetic acid (IAA) and siderophore production. The 16S rRNA sequence analysis of bacterial strains showed 98 to 100% identity with close relatives belonging to *Bacillus* and *Pseudomonas* genera. Greenhouse studies revealed that bacterial strains suppressed the *P. capsici* and significantly enhanced the plant growth characters in chilli pepper. These results confirmed the significant role of native rhizobacteria for the control of soil-borne oomycetes and the potential use of *Bacillus* and *Pseudomonas* spp. in bio-fertilizers and bio-fungicides development.
